# Male engagement in family planning: the role of faith leaders in urban West Africa

**DOI:** 10.1093/pubmed/fdad112

**Published:** 2023-07-17

**Authors:** Renske Hylkema, Onaedo Ilozumba

**Affiliations:** Athena Institute, Vrije Universiteit, Amsterdam, 1081 HV, the Netherlands; Institute of Applied Health Research, University of Birmingham, Edgbaston, Birmingham, B15 2TT, UK

**Keywords:** health promotion, public health, research

## Abstract

Sustainable Development Goal 3 aims to improve access to modern contraceptives and inform and educate people on family planning (FP). However, contraceptive use among women of reproductive age in West Africa is low at approximately 20%. One related factor is the limited engagement of males in FP decision-making. Addressing this issue requires a multiplicity of approaches, including the engagement of faith leaders. Faith leaders are often trusted by their congregants and could be an avenue to promoting male involvement in FP. In this report, we discuss the role of faith leaders in two West African countries, Nigeria and Ghana. We conducted 11 in-depth interviews with faith leaders in Nigeria and Ghana. Our exploratory findings indicate that faith leaders seem to have adequate knowledge and a positive perspective on male engagement and FP. In addition, the relationship of trust faith leaders maintain with their congregants is valuable in educating or counselling congregants on male engagement and FP.

## Introduction

The Sustainable Development Goal 3 aims to improve access to modern contraceptives and inform and educate people on family planning (FP).^1^^,^^2^ FP is a way for individuals or couples to monitor their pregnancies by using modern contraceptives .^2^ However, modern contraceptive use among women of reproductive age in West Africa is low at approximately 20%.^3^ Reasons for the low utilization include men’s desire for a large family,^4^ their disapproval of FP,^5^ and limited male engagement in FP decisions.^6^ Male engagement in FP could encourage couples’ use of contraception.^7–9^

A potential avenue to reach males is through faith leaders since there is extensive trust in them in West Africa.^10^^,^^11^ Their involvement in providing information on FP contributes to males’ perception of their engagement and communal decision-making in FP and increases modern contraceptive use.^12^^,^^13^ Furthermore, estimates project that by 2050 two thirds of the world’s population will live in urban areas.^14^ Given the long-term urbanization projections, low FP uptake in urban West Africa, and limited FP engagement of males in Ghana and Nigeria, we discuss the role faith leaders can play in male engagement in FP in urban West Africa.

### The current situation in Ghana and Nigeria

Ghana and Nigeria report high religion rates with over 85% of the population identifying as Christian or Muslim.^15^^,^^16^

In 2011 and 2012, Ghana’s and Nigeria’s government implemented policies, which provides FP services free of charge.^17^^,^^18^ As a result, the prevalence of contraceptive use raised to approximately 23% and 26% and the number of unintended pregnancies decreased.^19^ However, in Nigeria 14% (2012), and in Ghana 23% (2017) of pregnancies end in induced abortion. Both countries only permit abortions under strict circumstances, therefore illegal abortion rates remain high.^20–22^

### Methods

The first author conducted in-depth interviews on zoom with 11 faith leaders from Ghana and Nigeria (Table 1). All interviews were transcribed and analysed thematically using ATLAS.ti.^23^ We identified three themes related to our specific objective: 1) the trust relationship between faith leaders and their congregation; 2) faith leaders’ adequate knowledge and positive perspective on male engagement and FP; 3) faith leaders’ role as educators and counsellors in male engagement and FP.

**Table 1:** Participant details

**Participant**	**Age**	**Gender**	**Marital status**	**Church denomination**	**Country of residence**
1	60	Male	Married	**Restorationism**	Ghana
2	42	Male	Married	**Pentecostalism/Charismatic**	Nigeria
3	38	Male	Married	**Pentecostalism/Charismatic**	Ghana
4	32	Male	Single	**Roman Catholic Church**	Nigeria
5	Unknown	Male	Married	Pentecostal	Nigeria
6	38	Male	Married	**Reformed Churches**	Ghana
7	Unknown	Male	Married	**Pentecostalism/Charismatic**	Nigeria
8	40	Male	Married	**Pentecostalism/Charismatic**	Nigeria
9	38	Male	Married	**Pentecostalism/Charismatic**	Ghana
10	54	Female	Married	Non-denominational	Ghana
11	50	Male	Single	**Reformed Churches**	Ghana

## Results

### *The relationship* between faith leaders and congregants

Faith leaders are highly trusted by their congregants (Table 2 quote 1). However, a few faith leaders acknowledged that some of their congregants are reluctant to discuss sensitive topics such as abortions since most faith leaders see that as a sin. A few faith leaders perceived having authority over their congregants. They see that as an opportunity to influence congregants’ decision-making and mindsets (Table 2 quote 1).

**Table 2:** Participant quotes

*Quote 1*	*‘…… they share almost everything: marital issues, health issues, social challenges, yes, almost everything’ (Male, 50, Reformed Church).*
*Quote 2*	*So, in Africa, we have also gotten to the point where people listen to spiritual leaders, religious leaders. …… If you tell them something they try to do it. …… And I think religious leaders and other leaders in different circles are going to be great people, great tools, that can be used to change the mindset of people in order to live a good life (Male, 38, Pentecostal/Charismatic church).*
*Quote 3*	*‘…… for me, the man is the head of the house. He must be responsible for the outcome of whatever happens. So, as a man, you need to sit down with your partner and agree on what you want. Both of you need agreement on how many children you will have, based on your budget and your plan for the future. I think men are critically responsible for family planning’ (Male, age unknown, Pentecostal)*.
*Quote 4*	*We have something we call ‘home, health, education’. …… Home is all about family planning. …… So, it is a program that we do. We do it online, on air, we do it on TV, radio, everywhere. So, that we educate, not only attract one mass but the general public as well (Male, 60, Restoration church).*

### Faith leaders’ knowledge and perspective

Faith leaders considered themselves knowledgeable about methods like birth spacing and, or limiting the number of births by using contraceptives, such as condoms. Their knowledge might be attributable to governmental investments in FP programmes in both countries for more than ten years.^17^^,^^18^ Furthermore, faith leaders unanimously agreed that FP has religiously been approved and encouraged within their congregation. Besides, most faith leaders were in favour of modern contraceptives. In contradiction, research found that faith leaders are against the use of modern methods and prefer traditional methods or abstinence instead.^24^ However, their study was based on interviews mainly conducted with Catholic, and Muslim faith leaders, while our findings were based on interviews of which 91% of participants were protestant. This could explain the differences in their perceptions since the adoption of modern contraceptives could be influenced by faith. This aligns with a qualitative study in Uganda, which found that Catholics are more hesitant to adopt modern contraceptives.^25^

Additionally, faith leaders unanimously agreed that men need to be engaged in FP decision-making and reach a consensus by mutual decision-making. However, a few faith leaders particularly stressed men’s responsibility for FP. Therefore, in their narratives an underlying assumption remains that, due to the patriarchal society, the final decision in FP should rely on the men (Table 2 quote 3).

### Faith leaders’ role as educators and counsellors

Faith leaders stress the importance of FP and male engagement in counselling sessions, and FP education, sometimes in collaboration with health care professionals. This is shown effective in a previous study, where health care workers were used as promoters in faith-based health interventions.^26^ In addition, few faith leaders also spread FP messages outside of the church setting (Table 2 quote 4). Some faith leaders are institutionalizing FP and joint decision-making in FP through developing materials for health education as part of church practices.

## Conclusion

Faith leaders have opportunities to play an essential role in male engagement in FP. Their trust relationship with congregants is evident. Besides, their knowledge and positive attitude towards male engagement in FP have the potential to increase the knowledge and behaviour/mindsets of congregants and the general public. Their role could be substantive as counsellors and educators through promoting mutual decision-making in FP, and institutionalising male engagement in FP, which could back up their messages. However, barriers need to be taken into consideration, such as high fertility desires among men, and gendered dynamics, which could challenge faith leaders’ influence. The conclusions of this report should be made with caution considering the limited number of participants in our study. However, our findings show that by strengthening faith leaders’ role through collaboration with healthcare workers and broadening their target group outside of the church setting, they can contribute to increasing male engagement and improving FP uptake, which decreases child- and maternal mortality rates.

## Funding

Not applicable.

## Conflict of interest

The authors have declared that no competing interests exist.

## Data availability

The data presented in this study are available on request from the corresponding author. The data are not publicly available due to ethical and privacy reasons.

## Notes

**Renske Hylkema,** Researcher, Msc

**Onadeo Ilozumba,** Research Fellow, PhD
